# Accountability for maternal and newborn health: Why measuring and monitoring broader social, political, and health system determinants matters

**DOI:** 10.1371/journal.pone.0300429

**Published:** 2024-05-02

**Authors:** Jennifer Requejo, Allisyn C. Moran, Jean-Pierre Monet

**Affiliations:** 1 United Nations Children’s Fund, New York, New York, United States of America; 2 Johns Hopkins Bloomberg School of Public Health, Baltimore, Maryland, United States of America; 3 World Health Organization, Geneva, Switzerland; 4 United Nations Population Fund, New York, New York, United States of America; Public Library of Science, UNITED KINGDOM

## Abstract

This article offers four key lessons learned from a set of seven studies undertaken as part of the collection entitled, “Improving Maternal Health Measurement to Support Efforts toward Ending Preventable Maternal Mortality”. These papers were aimed at validating ten of the Ending Preventable Maternal Mortality initiative indicators that capture information on distal causes of maternal mortality. These ten indicators were selected through an inclusive consultative process, and the research designs adhere to global recommendations on conducting indicator validation studies. The findings of these papers are timely and relevant given growing recognition of the role of macro-level social, political, and economic factors in maternal and newborn survival. The four key lessons include: 1) Strengthen efforts to capture maternal and newborn health policies to enable global progress assessments while reducing multiple requests to countries for similar data; 2) Monitor indicator “bundles” to understand degree of policy implementation, inconsistencies between laws and practices, and responsiveness of policies to individual and community needs; 3) Promote regular monitoring of a holistic set of human resource metrics to understand how to effectively strengthen the maternal and newborn health workforce; and 4) Develop and disseminate clear guidance for countries on how to assess health system as well as broader social and political determinants of maternal and newborn health. These lessons are consistent with the Kirkland principles of focus, relevance, innovation, equity, global leadership, and country ownership. They stress the value of indicator sets to understand complex phenomenon related to maternal and newborn health, including small groupings of complementary indicators for measuring policy implementation and health workforce issues. They also stress the fundamental ethos that maternal and newborn health indicators should only be tracked if they can drive actions at global, regional, national, or sub-national levels that improve lives.

## Introduction

At the beginning of the Sustainable Development Goal (SDG) era [1], there was optimism about progress in maternal and newborn health. Trends showed substantive reductions in maternal and newborn mortality in the two decades leading up to 2015, although there were profound variations in progress across and within countries and a growing concentration of deaths among the most vulnerable population groups [2]. The SDG era also ushered in considerable political momentum around maternal and newborn survival. A road map for strategic actions to end preventable newborn mortality and stillbirths (Every newborn: an action plan to end preventable deaths or ENAP) was endorsed at the sixty-seventh World Health Assembly in 2014 [3], and the seminal report entitled, “Strategies toward ending preventable maternal mortality (EPMM)” [4]was released by the World Health Organization in 2015. The Every Woman Every Child Global Strategy for Women’s, Children’s, and Adolescents’ Health (2016–2030) [5]was also launched to translate relevant components of the SDG framework into concrete guidance on how to accelerate progress through a health systems and multi-sectoral approach.

Strong support for improving the measurement and monitoring of maternal and newborn health accompanied these launches to help hold the world to account for progress and to identify priorities for action. Examples of major measurement advancements achieved between 2015 and 2023 are the development of aligned indicators and targets for ENAP and EPMM [6], WHO’s convening of the Mother Newborn Information for Tracking Outcomes and Results (MoNITOR) [7] technical advisory group and development of an online toolkit with standardized metrics, the establishment of the Network for Improving Quality of Care for Maternal, Newborn, and Child Health (Quality of Care Network) [8], and the initiation of a project to revise the handbook on monitoring emergency obstetric care published in 2009 [9]. The WHO Standards for Improving Quality of Maternal and Newborn Care in Health Facilities was also published, which presents a theory of change showing how health system readiness influences health worker ability to provide respectful, evidence-based care that can, in turn, save maternal and newborn lives [10]. The WHO Standards includes a recommended set of quality of care indicators for monitoring purposes, which were elaborated and refined in a catalogue developed by the Quality of Care Network [8].

Conceptual approaches to maternal and newborn health also evolved during this time frame, further embracing the life course paradigm that emphasizes the importance of maternal-newborn service integration as well as women’s empowerment, gender equality, and experience of care for achieving global and national health goals [11,12]. The maternal and newborn exemplars project [13] similarly adopted a holistic framework positing that success in improving maternal and newborn survival results from progress on a combination of interrelated distal social and policy factors, intermediate health system dynamics, and proximal issues at the household and individual level [14]. Effective coverage cascades were also developed for select maternal and newborn health interventions to provide guidance on how to identify and address any bottlenecks in the areas of health service availability, accessibility, quality, and user adherence [15].

All these conceptual developments and measurement improvements re-affirm the 11 themes of EPMM, particularly principles concerning human rights, equity, and support for the mother-baby dyad [16]. The set of papers in this PLOS collection and summarized by Jolivet et al (placeholder) present findings from seven research studies aimed at validating ten of the EPMM indicators that capture information on distal causes of maternal mortality. These ten indicators were selected through an inclusive consultative process, and the research designs adhere to MoNITOR recommendations on conducting indicator validation studies [17,18]. Collectively they represent an important addition to the literature because of their focus on upstream determinants. Although there is growing recognition of the relevance of the broader social and policy environment on maternal and newborn health, most maternal and newborn health related measurement and research efforts in recent years have focused on reforming health systems and service quality improvement initiatives [19]. The rationale for incorporating indicators on distal determinants in the EPMM framework was originally premised on the view that epidemiologic and demographic transitions underway will result in an increasing proportion of maternal deaths from indirect causes rooted in the interplay of complex contextual factors [16]. However, the latest United Nations estimates show a slowdown in the pace of maternal mortality reduction and unevenness in the progression of these transitions [20]. Explanations provided for this slowdown point towards global and national crises (e.g., economic downturns, political instability, rising food insecurity, gender inequalities, climate change, and conflict induced displacement), indicating that regardless of a country’s demographic and epidemiological profile, examination of macro-level social, political, and economic factors is critical for understanding and accelerating progress in maternal and newborn survival. Hence, the main findings of these seven research studies carried out in India, Ghana, and Argentina remain highly relevant.

## Lessons learned

Four critical lessons learned from these studies are:

### 1) Strengthen efforts to capture maternal and newborn health policies to enable global progress assessments while reducing multiple requests to countries for similar data

Several of the studies [21,22] point out discrepancies between information on specific maternal health related policies reported to global mechanisms compared to national laws and statutes on record even when the same source documents are cited. These discrepancies reflect challenges with ensuring global policy databases are accurate and up-to-date rather than an indication of invalidity of the policy indicators themselves. The voluntary nature of country reporting on national policies, staff turnover impacting who completes survey instruments, variations in how policies are interpreted through different collection platforms, and temporal differences between when information is reported to the global level versus when information is examined at a country level may all explain discrepancies between information found in global policy databases compared to country records. WHO has captured maternal and newborn health related national policies since 2008 and has made methodological adjustments over time to improve the utility of its policy database (e.g., requesting countries to submit supporting policy documents and reviewing these documents against survey responses [23]).

Making country reporting on maternal and newborn health policies mandatory through Member State resolutions (World Health Assembly, UN General Assembly) or other international monitoring processes could improve the accuracy of global policy databases and enable robust multi-country analyses of relationships between policy environments, maternal and newborn health service utilization, and health outcomes. However, such steps come at the cost of increasing already heavy reporting burdens on countries and should only be done if there is clarity on how countries will benefit from the reporting. Any new global reporting requirements should be aligned with national monitoring and evaluation platforms and ideally come with investments in country information systems so that data collection and reporting is reliable, efficient, and in sync with country priorities. Consistent funding for maintaining and updating global policy databases is also essential for them to function as useful global public health goods.

### 2) Monitor indicator “bundles” to understand degree of policy implementation, inconsistencies between laws and practices, and responsiveness of policies to individual and community needs

Most of the seven studies found marked inconsistencies between existing national maternal and newborn health policies and actual practices by health providers and in health facilities. These findings confirm the adage that the existence of a policy, law or regulation does not guarantee its implementation. Having a policy or legal framework in place reflects country prioritization and provides the foundation for programme development and other actions. However, policy makers often have limited influence over how policies are implemented, and wide variation in practices are common across subnational areas especially in highly devolved health systems [24]. Hence, complementary indicators such as those that capture information on leadership as well as supply and demand side factors (e.g., financial flows, health system readiness including medicines and equipment, perceptions of service quality, experiences of discrimination, etc) that facilitate or impede policy implementation should be systematically tracked at national and subnational levels to ensure appropriate actions to address gaps. Odikro et al, for example, document vast differences across and within the three study countries on the extent to which actual practices mirror policies eliminating user fees for maternal health services [25]. They found multiple instances of women reporting that they made formal and informal payments for services that were supposed to be provided for free. These results are not proof of invalidity of the indicator that captures the legal status of user fees as part of country strategies to achieve universal health coverage. Instead, they show that the policy indicator on user fees should be monitored in combination with a set of indicators that capture implementation strength, including differentials by population groups and subnational region. The findings of the two articles by Gausman et al., on demand satisfied for family planning and on measuring SDG 5.6.2 (full and equal access to sexual and reproductive health care, information, and education) [26,27], consistent with the literature on gender equality and respectful care, show that indicators that capture women’s voices should also be regularly tracked so the development and implementation of maternal and newborn health policies reflect their needs [12,28].

### 3) Promote regular monitoring of a holistic set of human resource metrics to understand how to effectively strengthen the maternal and newborn health workforce

Three of the seven articles focus on indicators related to the midwifery workforce, highlighting mismatches between international reference standards (e.g., International Labour Organization’s international standard classification of occupations) compared to national competencies required for midwives, and discrepancies between what midwives are authorized to perform versus what services they typically provide in practice [22,29,30] The authors point out that these inconsistencies have serious implications for the accurate measurement and monitoring of core health workforce indicators around density and distribution, including assessments of progress towards the SDG index threshold of 4.45 doctors, nurses, and midwives per 1,000 population [1]. These study findings are consistent with documented challenges in capturing standard information on maternal and newborn health workforce cadres and skill levels through household surveys given enormous variation in cadres across countries and frequent changes over time in country lists of cadres and what they are permitted to do [31].

Achievement of universal coverage of maternal and newborn health services hinges upon the availability, accessibility, acceptability, and quality of the health workforce, including midwives, nurses, doctors, and community health care workers [32]. Yet, projections indicate there will be massive shortfalls in the health workforce by 2030 due to complex factors such as demographic and epidemiological transitions occurring at a different pace across countries, variations in educational policies determining the pool of qualified health workers, labor market dynamics affecting fair remuneration and equitable deployment, insufficient regulation of the private sector in many contexts, and inadequate enforcement of international agreements around health worker migration. The COVID19 pandemic shed light on other drivers impacting the health care workforce including safety (e.g., from infection, from attacks) and ensuring working conditions are conducive to health care providers being able to perform according to standards without the risk of burn-out. Research conducted prior to and during the pandemic also found that although frontline health care workers are disproportionately women, women are under-represented in leadership positions in the health care sector [33].

These factors combined call for greater accountability for achieving the ambitious milestones in the Global Strategy on Human Resources for Health adopted by the 69^th^ World Health Assembly in 2016 [32]. The Strategy includes a monitoring framework, and a minimum set of indicators capturing key workforce characteristics that are streamlined with reporting requirements for the SDGs and the WHO Global Code of Practice on the International Recruitment of Health Personnel. Greater investments are needed to support progressive implementation of National Health Workforce accounts and the associated WHO data portal designed to build country capacity to annually collect and report on workforce statistics including metrics on density, distribution, and performance. Such investments would help address the mismatches identified in the articles in this PLOS supplement and improve the quality and completeness of country health workforce data to the benefit of all including women and newborns. More research on the number of midwives required to ensure health facilities that provide childbirth services can be open 24 hours/seven days a week and the maximum number of births that a midwife or physician should manage per month would also help with setting appropriate density and distribution benchmarks. Complementary efforts that monitor the enabling environment for health care providers such as health facility assessments and studies on provider and patient experiences should also be regularly undertaken to generate a comprehensive picture of issues influencing health care worker ability to provide life-saving care.

### 4) Develop and disseminate clear guidance for countries on how to assess health system as well as broader social and political determinants of maternal and newborn health

The studies in this supplement viewed together also raise the important role of normative health agencies, working collaboratively with international health care professional associations, academic partners, country governments and other in-country partners, to generate and widely disseminate clear guidance on methods and approaches to assess health system functionality, health care worker competency levels, the quality of maternal and newborn care, and social and political determinants of maternal and newborn health. To support country reporting on the EPMM targets, for example, EPMM has developed some guidance materials on capturing evidence of the performance of signal functions and facility readiness measures. The ongoing project to revise the handbook on emergency obstetric and newborn care is an opportunity for the global community, working with country partners, to provide updated, comprehensive guidance on measuring and monitoring the availability and accessibility of emergency maternal and newborn health services as well as the quality of services provided. The WHO Quality of Care Network is another example of a broad-based partnership that develops guidance materials and other tools to support countries with the full cycle of monitoring, reviewing, and acting on evidence to improve the quality of maternal and newborn care. Similar guidance is needed to support country efforts to regularly monitor distal determinants and to triangulate data on these determinants with health system specific information to have a more comprehensive picture of current challenges and opportunities in maternal and newborn health.

A takeaway from the supplement articles and a principle of the Quality of Care Network and the Revisioning Emergency Obstetric and Newborn Care project is ensuring that countries are at the center of guidance development processes. Engagement of countries throughout the development process ensures the guidance addresses their needs and can be adapted to their contexts, thus increasing the likelihood of uptake. A mechanism should be in place to ensure that country experiences with implementing maternal and newborn health guidelines are shared and inform any guideline revisions.

## Conclusion

The indicator validation studies conducted as part of the Improving Maternal Health Measurement Project of EPMM published in this PLOS supplement make an important contribution to improving the measurement of upstream and intermediate determinants of maternal health, which are critical for achieving global and national goals. The study findings are consistent with ongoing maternal and newborn health measurement and monitoring efforts (e.g., ENAP-EPMM targets and integrated monitoring, the quality of care network, roll-out of the Maternal and Perinatal Death Surveillance and Response guidance, implementation of a protocol to improve measurement of skilled attendant at birth, etc.) as well as broader health system strengthening initiatives focused on supporting the health care workforce. We have distilled four key lessons learned from this collection of studies, all in keeping with the Kirkland principles of focus, relevance, innovation, equity, global leadership, and country ownership [34]. These lessons stress the value of indicator sets to understand complex phenomenon related to maternal and newborn health, including small groupings of complementary indicators for measuring policy implementation and health workforce issues. They also stress the fundamental ethos that maternal and newborn health indicators should only be tracked if they can drive actions at global, regional, national, or sub-national levels that improve lives.
